# First report of testis‐sparing surgery for sertoliform cystadenoma: case presentation and review of literature

**DOI:** 10.1002/iju5.12366

**Published:** 2021-09-07

**Authors:** Razvan‐George Rahota, Guillaume Ploussard, Jean‐Romain Gautier, Christophe Almeras, Henri Ducoin, Christophe Tollon, Jacques Assoun, Guillaume Loison, Jean‐Baptiste Beauval, Ambroise Salin

**Affiliations:** ^1^ Department of Urology La Croix du Sud Hospital Quint Fonsegrives France; ^2^ Department of Pathology La Croix du Sud Hospital Quint Fonsegrives France; ^3^ Department of Radiology La Croix du Sud Hospital Quint Fonsegrives France

**Keywords:** excisional biopsy, frozen section, sertoliform cystadenoma, testicular‐sparing surgery

## Abstract

**Introduction:**

Sertoliform cystadenoma is a very rare, benign lesion of the rete‐testis difficult to distinguish from other malignancies of the testicle.

**Case presentation:**

We present the case of a 42‐year‐old male who presented with a right testicular mass, asymptomatic for 1 year. Clinical examination revealed a palpable, painless, and well‐delimited right testicular superior pole nodule. Testicular ultrasound confirmed the nodule, whereas serum tumoral markers were normal. The patient underwent inguinal partial orchiectomy. Intraoperative excisional biopsy and frozen section pathology were performed, reporting undetermined tumoral origin with negative surgical margins. Ischemia time was 12 minutes. The final pathology report showed a Sertoliform cystadenoma of rete testis, with immunomorphology positive for AE1, CK7, and negative surgical margins.

**Conclusion:**

To our knowledge, this is the first report of testicular sparing surgery for Sertoliform cystadenoma, a very rare benign tumor of rete testis. All previously reported cases were managed by radical inguinal orchidectomy.

**Abbreviations & Acronyms**
AE1: Cytokeratin AE1
CAM5.2: Cytokeratin CAM5.2
CK7: Cytokeratin 7
CT: computed tomography
EMA: epithelial membrane antigen
PAX8: Paired‐box gene 8

**Keynote message**

To our knowledge, this is the first report of testis‐ sparing surgery for Sertoliform cystadenoma, a very rare benign tumor of the rete testis. All previously reported cases were managed by radical inguinal orchidectomy. Partial inguinal orchiectomy with frozen section pathology could be an alternative to radical surgery in selected cases.

## Introduction

Testicular tumors account for 1% of male cancers and approximately 5% of urogenital malignancies, with an increasing incidence due to the widespread use of ultrasound examination.^1^ Paratesticular tumors, especially masses of the rete testis, are a rare finding and can be classified in non‐neoplastic developmental pathologies (adenomatous hyperplasia, acquired or secondary cystic dysplasia), benign tumors (cystadenomas or Sertoliform cystadenoma), and malignant lesions such as adenocarcinoma of the rete testis.^2^, ^3^ The first report in the literature of Sertoliform cystadenoma was made by Jones MA in 1997.^4^ All previously reported cases of sertoliform cystadenoma have been managed by radical surgery‐ inguinal orchiectomy.^5^, ^6^ We present the first case of a Sertoliform cystadenoma in a 42‐year‐old male managed by conservative surgery.

## Case report

**Fig. 1:**
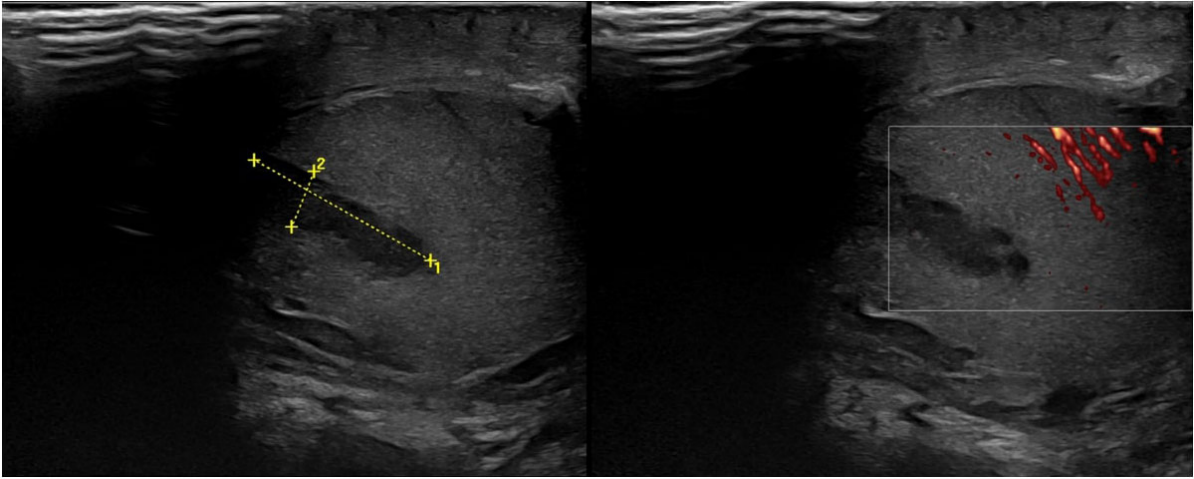
Scrotal ultrasound showing a 1.7/0.5 cm mass in the upper pole of the right testicle

A 42‐year‐old male with no prior medical history presented with a palpable mass in the right testicle, asymptomatic, and without any increase in size for 1 year. The patient is married, without children. At clinical examination, a firm, painless, well‐delimited superior pole right testicular lesion was objectified. Scrotal ultrasound revealed a 1.7/ 0.5 cm mass in the upper pole of the right testicle, with normal contralateral testicle and normal testicular volumes (Fig. 1). Serum tumor markers were collected and in normal range, as following: alpha‐fetoprotein (0.5 ng/ml, *N* = 0.89‐ 8.78), beta‐human chorionic gonadotropin (0, *N* < 1.4 UI/L), and lactate dehydrogenase (10 UI/L, *N* = 120–280 UI/L). A staging chest–abdominal–pelvic contrast‐enhanced CT scan was performed which did not show any evidence of visceral or lymph node metastasis.

**Fig. 2:**
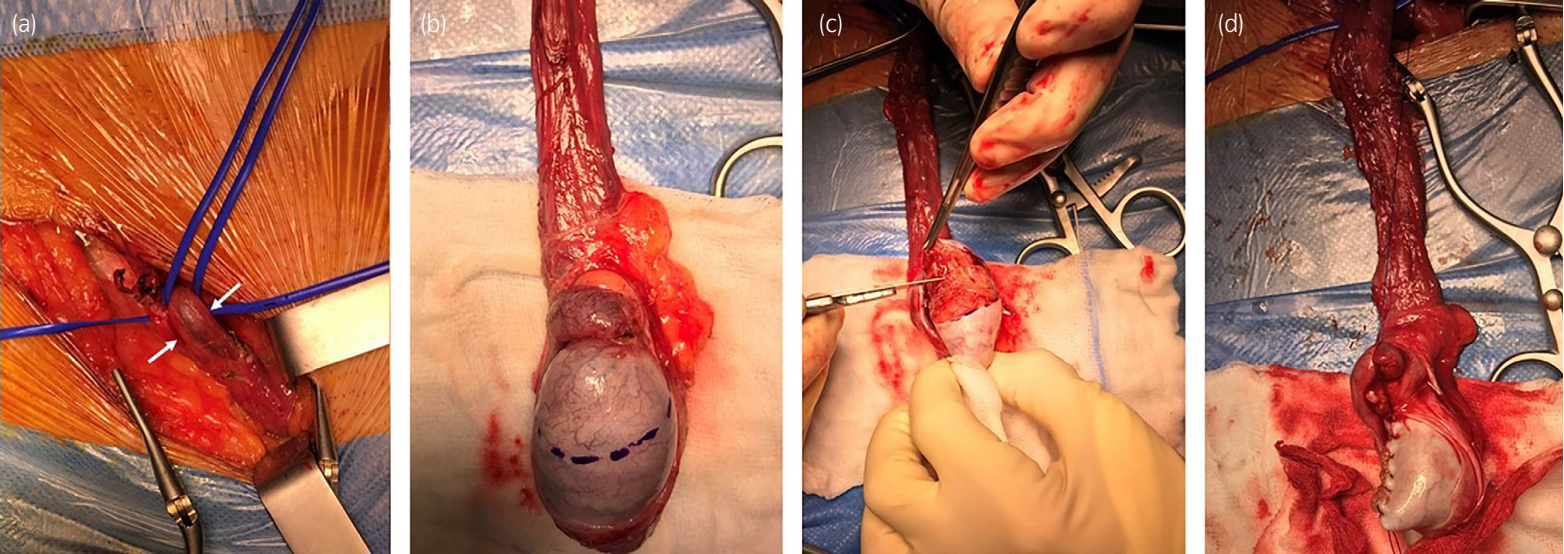
(a) Dissection of vas deferens (white arrow) and spermatic vessels (yellow arrow), (b) lesion identification and (c) excision, (d) final aspect after closing the albuginea

Given the fact that the lesion was perceived as stable by the patient for 1 year, as described during the first consultation, we suspected that a benign etiology could be possible and offered the patient the option of testis‐sparing surgery. The patient was informed of the risk of radical orchiectomy in case of intraoperative aspect or frozen section pathology report highly suggestive for malignancy and salvage radical orchiectomy if the final pathology report would be conclusive for malignancy. After informed consent was given, the patient underwent right partial orchiectomy by inguinal approach. We identified and dissected the elements of the spermatic cord, after which we separated the vas deferens from the testicular vessels. After clamping the testicular vessels, we incised the tunica albuginea and proceeded with the partial orchiectomy within safety surgical margins. Frozen section assessment was demanded and revealed testicular tumor of indeterminate origin, with negative surgical margins. Thus, we closed the albuginea and reintegrated the ipsilateral testicle in the scrotum, at the same time performing an orchidopexy (Fig. 2). The total ischemia time was 12 min.

**Fig. 3:**
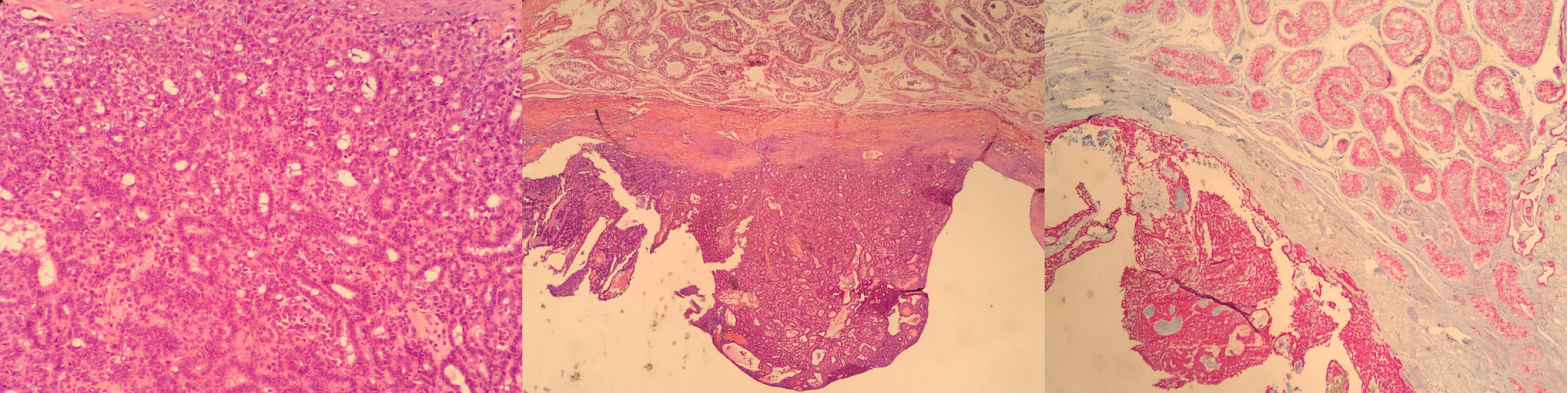
Morphology aspect of well‐formed tubules and poorly defined lamina suggestive of Sertoliform cystadenoma

The final pathology report showed a Sertoliform cystadenoma of the rete testis, with the tubular architecture of variable sizes, without atypical cells and rare mitosis visible. The testicular parenchyma present on the specimen was without pathological characteristics and the surgical margins were negative. Immunohistochemistry was intense positive for AE1 and CK7, negative for Inhibin and EMA, without any signs of malignancy (Fig. 3).

The patient was discharged the same day, no immediate or long‐term postoperative complications were reported. The 1‐month postoperative scrotal ultrasound examination was normal, with no sign of local recurrence. Due to the fact that it is a rare histology, we performed also 2 and 6‐months postoperative serum tumoral markers, which were all negative.

## Discussion

Sertoliform cystadenoma is a rare benign tumor of the rete testis, being for the first time mentioned in the literature in 1997 by Jones *et␣al*.^4^ The largest case series in the literature was presented by Paluru *et␣al*.^6^ and is comprised of 15 cases; mean age reported was 46 years, mean tumor size 1.5 cm, mean follow‐up of 8.1 years with no complications and no evidence of disease recurrence. All of the patients underwent radical inguinal orchidectomy.

Several single case reports have been also identified in the literature. Sahnan *et␣al*. and Bremmer *et␣al*. describe the cases of a 19 and 66‐year‐old man, respectively, with a 6‐month history of smooth, painless right testicular mass, stable over time, with normal serum tumoral markers in which right radical inguinal orchidectomies were performed.^7^, ^8^

The first pediatric case was reported by Kacar *et␣al*.^9^ in a 6‐year‐old boy who presented bilateral gynecomastia and left scrotal tumefaction. Serum tumoral markers were in the normal range but estradiol and testosterone levels were slightly increased (48.4 ng/mL and 20 ng/dL, respectively). No other adult patient reported in the literature displayed increased levels of sexual hormones. The patient underwent left inguinal orchidectomy and the final pathology report revealed Sertoli cell proliferations, compatible with Sertoliform cystadenoma.

The most recent case of Sertoliform cystadenoma was presented by Lahouti *et␣al*. in a 39‐year‐old male with a right superior pole testicular mass,^5^ in which serum tumor markers were negative. The final pathology report revealed sertoliform cystadenoma with immunohistochemical examination intense positive for Inhibin, cytokeratin CAM5.2, PAX8.

Histologically, the most important differential diagnosis is made with rete testis adenocarcinoma. Sertoliform cystadenoma has been reported in patients between 19 and 84 years, having variable size characteristics (a few mm to 4 cm) with a predominant tubular architecture, columnar to cuboidal tumor cells with eosinophilic cytoplasm, and basally located nucleus. Moreover, adenocarcinoma of rete testis is also a rare but aggressive finding, with an incidence peak around 70 years of age. The most important morphologic criteria required for diagnosis are the involvement of testicular mediastinum with the preservation of the parietal tunica vaginalis, in the absence of other lesions.^5^, ^10^

Although current recommendations are in favor of considering every scrotal mass as malignant until proven otherwise, we propose in selected cases where clinical history is in favor of benign lesion and patients desire maximum fertility preservation the alternative of organ sparing surgery. Testis‐preserving surgery should always be accompanied by frozen section examination, due to the fact that it has a high concordance with the final pathology result.^11^ However, all patients must be informed of the risk of immediate or salvage radical orchidectomy if the frozen section or final pathology report are consistent with malignancy.

## Conclusions

In conclusion, we report the first case of Sertoliform cystadenoma managed by conservative surgery—partial orchidectomy, without any early or late postoperative complications. Sertoliform cystadenoma remains a rare finding among intratesticular mass findings, with only 16 cases reported in the literature, all treated by radical inguinal orchidectomy.

## Funding source

No funding was received for this study.

## Conflict of interest

The authors disclose no conflicts of interest.

## Approval of the research protocol by an Institutional Reviewer Board

This study was approved by the local ethics committee of the Croix du Sud hospital (register number 57/2021) and was conducted in line with the principles of the Declaration of Helsinki.

## Informed Consent

Not applicable.

## Registry and the Registration No. of the study/trial

Not applicable.

## References

[1] Albers P , Albrecht W , Algaba F *et␣al*. Update. Eur. Urol. 2015; 2015(68): 1054–68. 10.1016/j.eururo.2015.07.044 26297604

[2] Emerson RE , Ulbright TM . Morphological approach to tumours of the testis and paratestis. J. Clin. Pathol. 2007; 60: 866–80. 17307866 10.1136/jcp.2005.036475 PMC1994505

[3] Rosevear HM , Mishail A , Sheynkin Y , Wald M . Unusual scrotal pathology: an overview. Nat. Rev. Urol. 2009; 6: 491–500. 19668251 10.1038/nrurol.2009.149

[4] Jones MA , Young RH . Sertoliform rete cystadenoma: a report of two cases. J. Urol. Pathol. 1997; 7: 47–53.

[5] Lahouti AH , Brodherson M , Larish Y , Unger PD . Sertoliform cystadenoma of the rete testis: report of a case and review of the literature. Int. J. Surg. Pathol. 2017; 25: 555–8. 28413913 10.1177/1066896917704304

[6] Paluru S , Ulbright TM , Amin M , Montironi R , Epstein JI . The morphologic spectrum of sertoliform cystadenoma of the rete testis: a series of 15 cases. Am. J. Surg. Pathol. 2018; 42: 141–9. 29240582 10.1097/PAS.0000000000000997

[7] Sahnan K , Manjunath A , Vaughan‐Shaw PG , Mitsopoulos G . An unexpected finding of a rare intrascrotal lesion: the sertoliform cystadenoma of the rete testis. Case Rep. 2013; 2013(feb20 1): bcr2012008439. 10.1136/bcr-2012-008439 PMC3604416 23429025

[8] Bremmer F , Schweyer S , Behnes CL , Blech M , Radzun HJ . Sertoliform cystadenoma: a rare benign tumour of the rete testis. Diagn. Pathol. 2013; 14(8): 23. 10.1186/1746-1596-8-23 PMC3584937 23406299

[9] Kacar A , Senel E , Caliskan D , Demirel F , Tiryaki T . Sertoliform cystadenoma:\x92a case with overlapping features. Pediatr. Dev. Pathol. 2011; 14: 138–43. 20658931 10.2350/10-01-0778-CR.1

[10] Lopez‐Beltran A , Canas‐Marques R , Raspollini MR , Montironi R , Scarpelli M , Cheng L . Cysts and Epithelial Proliferations of the Testicular Collecting System. In: Colecchia M (ed). Pathology of Testicular and Penile Neoplasms. Springer, Berlin, Germany, 2016.

[11] Matei DV , Vartolomei MD , Renne G *et␣al*. Reliability of frozen section examination in a large cohort of testicular masses: what did we learn? Clin. Genitourin. Cancer 2017; 15: e689–96. 28216275 10.1016/j.clgc.2017.01.012

